# Report of a Case of Laparotomy for Gun-shot Wound of the Small Intestine and Mesentery

**Published:** 1885-11

**Authors:** D. A. V. Park

**Affiliations:** Chicago


### REPORT OF A CASE OF LAPAROTOMY FOR GUN-SHOT WOUND OF THE SMALL INTESTINE AND MESENTERY. *By* D. A. V. PARK, M. D., CHICAGO.

[Read before the Chicago Medical Society, 5th October, 1885.]

The case I have to present is that of M. S., a young butcher boy, sixteen years old, slight in stature, and of under size; having the appearance of a lad fourteen years of age, rather than of sixteen years. His health for the past two years has been good, previous to that, quite delicate, and to such a degree, that he could not run and play with his young companions.

Sept. 1st, 1885, 3:30 P. M., while picking apples in an orchard, was fired upon by the manager and wounded in the abdomen. The boy threw up his hands and cried out to a bevy of his young companions, saying, "Boys, I am shot!" The manager was standing forty-five feet away; — the size of pistol ball, 22 calibre; there was profuse hæmorrhage from the wound externally, while the boy was standing; as soon as he was persuaded to lie upon the ground all external hæmorrhage ceased. While at Eighty-ninth street, a physician saw the boy and examined the wound with a probe; he could not pass the probe into the abdominal cavity, and said the boy's chances for recovery were good. The boy was removed from Eighty-ninth street to his home, the corner of Thirty-first and Wallace streets, a distance of something more than seven miles. A farmer's half-spring, light wagon was used as an ambulance; reached home 7:30 P. M. I saw the boy at eight o'clock P. M.

Upon a careful examination, I observed the following symptoms, viz: voice feeble, but could converse audibly and in a coherent and intelligent manner; face and lips colorless; extremities cold (shock slight); temperature 99° F., pulse 100, respiration 24, vomiting continuously, thirst great. Examination of abdomen by immediate percussion, the following physical conditions were noticed: marked tenderness over entire abdomen, abdomen tympanitic and walls rigid. Pistol ball entered abdominal wall at a point mid-way between symphysis pubis and umbilicus, and two inches to the left of the median line. Probed wound, a fine, flexible silver probe being used; could trace the course of the ball through skin, adipose and muscular tissue, to peritoneum, and thence downward one inch; could not trace ball through peritoneum into abdominal cavity. Dressed wound with iodoform and carbolized cotton gauze; as light a dressing as possible; gave morphia hypodermically, one-fourth grain; directed nurse how to apply cold applications to abdomen, and to give morphia, in powders containing one-fourth grain each, every three or four hours, if required.

11 o'clock P. M. Patient slept soundly for two hours. Extremities warm; but little pain; no vomiting; great thirst; nurse gave water with teaspoon; gave morphia, one-fourth grain.

3 o'clock P. M. Patient rested well for four hours. Awoke; got out of bed; walked out into sitting-room; helped himself to a glass of cold water, voided urine, not without great pain and considerable difficulty; character of urine, normal; nurse gave morphia, one-fourth grain.

5 o'clock A. M. Patient rested for two hours, awoke, wanted to know what time it was, and asked if he could not dress himself and go to work, this being the usual hour for going to work; drank more water and was about to dress himself, when attacked with a severe and protracted fit of vomiting; complained of severe pain in abdomen and limbs.

8 o'clock A. M. Patient more restless, more pain and vomiting; temperature 100° F., pulse 125, respiration 24; gave morphia hypodermically, one-fourth grain; thirst great.

10 o'clock A. M. Patient resting quietly; vomiting ceased; temperature, pulse and respiration same as at eight o'clock; no pain.

Dr. D. A. K. STEELE saw patient with me, made a careful examination, verified my diagnosis, and we decided to operate at once. To do laparotomy in a filthy room of small dimensions, with two typhoid patients in rooms adjoining, and a father on a protracted debauch, making both day and night hideous with his drunken revelry, were anything but pleasing inducements to the surgeons contemplating an operation of such magnitude. After due consideration, we decided to remove my patient to the Michael Reese Hospital. By assuring him that his chances for recovery would be far better at the hospital than at his home, by impressing the importance of this fact upon the minds of a brother and sister, they gave their consent to the removal.

12 o'clock M. Patient resting quietly; temperature 100° F., pulse 125–30, respiration 24; conscious. Removed patient on a stretcher in a patrol wagon to hospital, being protected from the scorching sun by an umbrella.

1 o'clock P. M. Patient arrived at hospital nearly exhausted. Temperature 100° F., pulse 130, weak and intermitting, respiration 30; abdomen tympanitic; tympanitic resonance extending on left side as high as hypochondriac region, and this to such a degree that no characteristic liver dullness could be obtained, and we believed that extravasation of bood into abdominal cavity had taken place to such an extent that this organ was crowded upward to a marked degree. We are taught by no less authority than the late PROFESSOR GROSS that no surgical operation of any magnitude should ever be undertaken without due consideration and careful preparation; remembering this, the physicians and surgeons of hospital staff who were present, Dr. Steele and myself, repaired to a room and counseled together as to the advisability and the practicability of operating, twenty-two hours having elapsed since injury occurred.

We concluded to operate, and at once.

Patient was placed on operating table; abdomen shaved and washed with a 2 per cent. carbolized solution; legs wrapped from toes to hips with batting, made fast with a roller bandage; patient anæsthetized; ether from a common towel was the agent exhibited. Patient catheterized, and one-half pint of normal urine obtained.

1:30 P. M. Operation: An incision four inches long was made two inches to the left and on a line with the linea alba and directly over seat of wound, direction of wound followed as the incisions were made through skin, adipose and muscular tissue to peritoneum, care taken to secure bleeding vessels as we divided each tissue; could not find where the ball passed through peritoneum or discover wound thereof. The thin tendinous layer of subperitoneal fascia was picked up with tissue forceps, lifted up and clipped with scissors and slit up on a director; as peritoneum was raised and clipped in the same manner, decomposed blood rushed through opening thus made with great force, blood and blood clots which quickly formed were removed with sponges, intestine drawn out of cavity and examined for wound. First wound found was an abrasion. Ball had carried away peritoneal and muscular coats; did not penetrate mucous coat.

But little hæmorrhage; wound closed up by the interrupted catgut suture. Second wound, one-half inch in diameter, opened directly into the intestinal cavity, thence through mesentery near its junction with intestine. A small mesenteric artery was found divided and from which hæmorrhage was taking place. Artery was tied; all hæmorrhage ceased; wound in intestine closed by the interrupted suture. "The fine catgut sutures were introduced about one-third of an inch from the divided edges, made to enclose the peritoneal and muscular coats and brought out just free of the edge on one side, and were then re-introduced close to the edge and made to enclose the same amount and kind of tissue on the other side; great care taken to include muscular coat and not to allow the needle to pass into the intestinal cavity; edges of the mucous membrane were turned in and the sutures tightened, thus two broad surfaces of peritoneum were brought together." We could not detect any further injury. Abdominal cavity was cleansed thoroughly with a 1 per cent. solution of carbolized water, intestines cleansed with a solution of same strength and returned to cavity, being carefully examined as they were being returned. Intestines were protected and kept warm with large muslin cloths wrung out of warm water, during their removal from cavity. The intestines were normally distended

Abdominal incision was closed by two sets of sutures, the peritoneal surfaces approximated and closed by the continuous suture; skin, adipose and muscular walls closed by interrupted suture and adhesive strips applied. Cotton gauze and roller bandage applied; dressing made as light as possible. At close of operation patient was assigned to ward B, bed 37, enveloped in heavy woolen blankets and bottles of hot water applied to feet, legs and trunk of the body.

4 o'clock P. M. Temperature 100° F., pulse 160, soft and rapid, could be counted with difficulty, respiration 47; quite thirsty; gave an enema of brandy and water. Patient soon began to perspire freely, and after an hour one of the blankets was removed.

5:30. Temperature 105°.5 F., pulse 140, respiration 40. Patient got out of bed on his hands and knees on to the floor; after this he was tied in bed; pulse improved in strength, and at this time pulse was quite strong and regular, and the boy's chances appeared improved.

6 o'clock. Another enema of brandy and water administered.

At 6:30 commenced giving a teaspoonful or two of a mixture composed of equal parts of milk and lime water every half hour.

7 o'clock. Pulse had less force, was more rapid and irregular; two hypodermics of brandy given, with a somewhat favorable effect on the pulse. Temperature 101° F., pulse 150, respiration 44.

8 o'clock P. M. A third enema of brandy and water given; pulse still rapid and weak. At 8:30, 9 to 9:30 pulse continued rapid, weak and irregular, in spite of several hypodermics of brandy and ammonia administered at the corresponding hours. Cardiac impulse strong and angry.

10 o'clock P. M. Enema of brandy and milk (1 to 4) given; boy still delirious and attempted to release himself; skin no longer moist.

10:30 P. M. Notwithstanding his restraint, patient got a glass half full of water from his stand and drank it, causing profuse vomiting of the liquid contents of the stomach and some bile; urinated in bed. Bedding changed and more bottles of hot water added and continued all night. Milk and lime water advised to be discontinued.

11 P. M. Pulse continues about the same, weak and rapid; appearance of countenance anxious, restless, otherwise bright.

12 M. Enema of brandy and milk. Difficult to count the pulse.

12:30 Enema of brandy and milk.

2 A. M. Vomited again.

2:30 Enema of brandy and milk.

3:30. Vomited a third time. Involuntary defecation.

5 A. M. Legs and feet getting cold. Hands and face cold and covered with a clammy perspiration; breathing hurried; pulse exceedingly rapid; more bottles of hot water advised and hypodermics of brandy administered; hands become colder; muscles of eyes paralyzed; breathing becomes more labored. Radial pulse imperceptible; respiration sighing and infrequent; dropping of the chin, and at 5:15 A. M. patient died.

Phenomena observed during operation were as follows, viz.: a large quantity of blood in abdominal cavity. The rapidity with which it became coagulated when atmospheric air was admitted into abdominal cavity. The hæmorrhage from small vessels and contusions ceased almost instantly on exposure to air. Extravasation of intestinal contents so little that it could not be detected. Intestines empty or free from usual contents. Under the influence of the anæsthetic, pulse grew strong and regular; this continued until operation was nearly finished when pulse grew rapid and weak, and to such a degree that hypodermic injections of brandy were given often.

12:30 P. M. A post mortem examination revealed commencing peritonitis, intestines being agglutinated together, and this appeared to be quite general through entire mass of small intestines; a quantity of extravasated blood and a few blood clots were found in the upper part of left lumbar and lower half of left hypogastric regions; at the seat of wound in ileum, where the peritoneal surfaces were drawn together with the interrupted ligatures, little bands of exudate had passed from side to side, and in one place to such an extent as to cover the knot in the ligature, thus showing that the work of repair had advanced to a marked degree; a contused wound of rectum was found near its junction with sigmoid flexure; the ball striking this point was evidently deflected downward and outward, as it was found imbedded in muscular tissue just beneath and posterior to Poupart's ligament, and but an inch or two from the point at which the boy located the position of ball, while being examined; his remark was like this: "I felt the ball stop right there, just at that point," indicating the place by the finger.

In this report, I have tried to outline as briefly as possible the points of interest from the time the boy received the wound to the autopsy, interesting in a surgical, also of great interest in a diagnostic point of view; to the surgeon surgical interference was imperative; it would be difficult to conceive of a more favorable subject: a young boy just developing into manhood, with great tenacity for life, and who had not partaken of food for ten hours previous to receiving wound, thus explaining the entire absence of extravasation of intestinal contents into abdominal cavity, this being so slight that it could not be detected. The wound to intestines was not great, the small size of blood vessel injured, the ball imbedded in muscular tissue where it would become encysted: thus summing up the amount and kind of injury to abdominal contents, I am sanguine in the belief, had it been possible to have done laparotomy immediately, with all necessary antiseptic precaution, that our earnest labor would have been crowned with the rich honors and reward that await the conscientious, skillful surgeon in this comparatively new field of surgical science.

This case corroborates the statement so often made by eminent surgeons, that no reliable knowledge as to direction or course of a bullet can be obtained from position of a wound of entrance or of exit, if one there be. I recall a number of cases that are also illustrative of this fact, two of which I will note,—the first coming under my observation last January; the second occurred during the war of the rebellion. J. E., 50 years old, saloon-keeper, received shot from a Smith & Wesson revolver, 32 calibre; distance not over six feet. Bullet entered the left shoulder two inches above axilla, passing through deltoid muscle. The shock from wound was great, the man falling heavily to the floor, extremities paralyzed. Examined the man; saw wound in shoulder; could find no wound of exit. Passed hand gently along the back and found what I believed to be the bullet, two inches to the right of spinal column and on a line with spinous process of tenth dorsal vertebra. Made incision through skin and adipose tissue and removed bullet. Patient survived his wound but twenty-two hours. Post mortem: found that bullet passed directly upward, striking clavicle at the point of deltoid attachment; deflected downward and backward, passing through upper and anterior portion of superior lobe of lung; thence downward through posterior and lower portion of inferior lobe of left lung, passing directly through the body of the tenth dorsal vertebra and two inches to the right thereof, being the point where found.

W. P., Company I, 68th Ohio Volunteer Infantry, at the battle of Fort Craig, was hit by a minie ball, it striking the clasp on his belt; deflected from this, passed through leather belt, clothing, and just beneath the skin, near the median line, encircled one-half of the body, point of exit being at spinous process of dorsal vertebra. The shock was so great that the soldier did not recover consciousness for some time. He was left upon the field by the surgeon to die; being removed to hospital the following day, it was discovered that bullet had not penetrated abdominal cavity. It is needless to say that the soldier made a rapid and easy recovery.

From the rich experience derived from this case of laparotomy, I should favor making an " extensive incision, freely exposing the abdominal contents, as well as cavity, in order that all the viscera might be thoroughly and carefully exposed, and every wound brought within reach."

In a masterly paper by our honored President, entitled " Gun-shot Wounds of the Small Intestines," and read by him at the meeting of the American Medical Association, 1884, he wrote these lines:

" A period of one hundred years and more has rolled away since Dr. Heister published his belief and reported recoveries, to the time when Dr. Sims expresses his convictions—over a century of doubts, timidity, uncertainty and gloomy misgivings, lightened only occasionally by some bold and resolute assertions. The future asks for action, and it is not unreasonable to assert that careful trials will accomplish successful results."

" Avoiding any spirit of dictation, it seems proper to tabulate the following conclusions as an outgrowth of the experiments:

" 1st. Hæmorrhage following gun-shot wounds of the abdomen and the intestines is very often so severe that it cannot be safely controlled without abdominal section; it is always sufficient in amount to endanger life by secondary septic decomposition, which cannot be avoided in any other way than by the same treatment.

" 2d. Extravasations of the contents of the bowel after shot injuries thereof are as certain as the existence of the wound.

" 3d. No reliable inference as to the course of a bullet can be made from the position of the wounds of entrance and exit.

"4th. The wounds of entrance and exit of the bullet should not be disturbed in any manner, except to control bleeding or to remove foreign bodies when present. They need only to be covered by the general antiseptic dressing applied to the abdomen.

"5th. Several perforations of the intestines close together require a single resection, including all the openings. Wounds destroying the mesenteric surface of the bowel always require resection.

"6th. The best means of uniting the wounded intestine after resection is by the use of fine silk thread, after Lembert's method. It must include at least one-third of an inch of bowel tissue, passing through only the peritoneal and muscular coats, never including the mucous coat. The everted mucous membrane must be carefully inverted, and needs no other treatment.

"7. Wounds of the stomach, small perforations and abrasions of the intestine, can be safely trusted to the continued catgut suture.

"8th. Every bleeding point must be ligated or cauterized, and especial care devoted to securing an absolutely clean cavity.

"9th. The best method of treating the stumps of divided mesentery is to save the mesenteric surface of the bowel as above indicated.

"10th. Primary abdominal section in the mid-line gives the best command over the damage done, and furnishes the most feasible opening through which the proper surgical treatment of such damage can be instituted. Further, its adoption adds but little, if anything, to the peril of the injury.

"11th. Is not the moral effect of the assurance to the patient that he will be placed in a condition likely to lead to his recovery, a good substitute for the mental depression accompanying the general and popular conviction that these wounds mean certain death ?"

Again, would it not be eminently practical to make an exploratory incision, in order to verify diagnosis, and to enlarge this incision to a complete abdominal section, for no less heroic procedure will accomplish successful results? The importance of complete abdominal section is apparent when it is remembered that the autopsy in this case revealed a large quantity of extravasated blood and a few blood-clots within the abdominal cavity.

From a scholarly paper by Professor Norman Bridge, entitled "The New Science of Medicine," I quote the following:

"The expression abdominal surgery has a meaning that is entirely modern. To have learned that the abdominal cavity may be opened with comparative safety, and to have done ovariotomy thousands of times, as well as other formidable operations upon the abdomen, with a large majority of successful results, would appear to be glory enough for a century. But the splendor of the achievements cannot be appreciated till we attempt to compute the saving of life in years."

In conclusion, I desire to publicly announce my earnest and sincere thanks to the following gentlemen, who so kindly assisted me in my vain effort to alleviate the suffering and to save the life of a fellow being, viz: Dr. Henry Banga, Dr. Edward Kuh, Dr. R. G. Collins, Dr. L. E. Frankenthal, of the Michael Reese Hospital; to Professor D. A. K. Steele for earnest interest taken and skillful assistance rendered.

